# Risk factors for brain metastases in completely resected small cell lung cancer: a retrospective study to identify patients most likely to benefit from prophylactic cranial irradiation

**DOI:** 10.1186/1748-717X-9-216

**Published:** 2014-09-20

**Authors:** Hui Zhu, Yongmin Bi, Anqin Han, Jingyu Luo, Minghuan Li, Fang Shi, Li Kong, Jinming Yu

**Affiliations:** Department of Radiation Oncology, Shandong Cancer Hospital and Institute, Shandong University, Jiyan Rd. 440, Jinan, 250117 Shandong Province China; Department of Radiology, the Airforce General Hospital of Chinese People’s Liberation Army, Beijing, China; Department of Thoracic Surgery, Shandong Cancer Hospital and Institute, Jinan, Shandong Province China

**Keywords:** Small cell lung cancer, Brain metastases, Prophylactic cranial irradiation, Risk factors, Lymphovascular invasion, Pathologic stage

## Abstract

**Background:**

The role of prophylactic cranial irradiation (PCI) on small cell lung cancer (SCLC) has been established based on the two-stage system of limited versus extensive disease and the treatment modality of chemoradiotherapy. However, the use of PCI after combined-modality treatment with surgery for resectable limited-stage SCLC has not been investigated sufficiently. We conducted a retrospective study to evaluate risk factors for brain metastasis (BM) in patients with surgically resected SCLC to identify those most likely to benefit from PCI.

**Patients and methods:**

The records of 126 patients with completely resected SCLC and definitive TNM stage based on histological examination between 2003 and 2009 were reviewed. The cumulative incidence of BM was estimated using the Kaplan–Meier method and differences between the groups were analyzed using the log-rank test. Multivariate Cox regression analysis was applied to assess the risk factors of BM.

**Results:**

Twenty-eight patients (22.2%) developed BM at some point during their clinical course. The actuarial risk of developing BM at 3 years was 9.7% in patients with p-stage I disease, 18.5% in patients with p-stage II disease, and 35.4% in patients with p-stage III disease (*p* = 0.013). The actuarial risk of developing BM at 3 years in patients with LVI was 39.9% compared to 17.5% in patients without LVI (*p* = 0.003). Multivariate analysis identified pathologic stage (hazard ratio [HR] = 2.013, *p* = 0.017) and LVI (HR = 1.924, *p* = 0.039) as independent factors related to increased risk of developing BM.

**Conclusion:**

Patients with completely resected p-stage II-III SCLC and LVI are at the highest risk for BM.

## Background

Small cell lung cancer (SCLC) accounts for approximately 15%–20% of all lung cancers [1]. It is characterized by a high incidence of metastatic disease at presentation, rapid doubling time, and a high response rate to treatment [2]. Despite a high response to chemotherapy (ChT) and radiation therapy, most patients suffer from local recurrence or/and distant metastasis within 2 years. Brain metastases (BM) are the most common site of distant failure in patients with SCLC, regardless of disease stage at presentation. The prevalence of BM detected at the time of diagnosis ranges from 10% to 24% and the probability of developing BM during the course of disease increases to 50% by 2 years after diagnosis. BM is associated with a poor prognosis and the median survival after the development of BM is only 4–6 months [3–5].

The survival advantage conferred by prophylactic cranial irradiation (PCI) in patients with SCLC has been established in recent studies. As a result, PCI has become part of the standard treatment modality for patients with SCLC who had stable disease or a better response to ChT, with or without thoracic radiation therapy (TRT) [6–9]. The positive role of PCI on patients with SCLC was based on the two-stage system and treatment modalities of non-surgery. But the incidence of brain metastases in surgically treated SCLC and the risk factors for developing BM in this subgroup were rarely evaluated.

We reviewed the patients with completely resected SCLC and assessed the possible risk factors for developing BM in this patient population.

## Patients and materials

### Study population

We retrospectively reviewed the records of patients with completely resected SCLC and definitive TNM stage on the basis of histological examination at the Shandong Cancer Hospital and Institute between January 2003 and December 2009. All patients underwent a standardized evaluation, including thoracic and abdominal computed tomography scanning or abdominal ultrasonography, brain magnetic resonance imaging, and bone radionuclide imaging before surgery. All patients had negative brain computed tomography (CT) scan or magnetic resonance imaging (MRI) preoperatively and PCI was not conducted to them postoperatively. During this period, 211 patients with SCLC were operated in our cancer center, and 18 patients of them who had R1, R2 resections were excluded in this study in order to avoid bias of the results, sixty-seven patients were given PCI, so a total of 126 patients met the criteria. The study was approved by the institutional review board and ethics committee at the Shandong Cancer Hospital and Institute.

### Treatment

Surgical procedures included lobectomy or pneumonectomy with ipsilateral hilar and mediastinal lymphadenectomy. Patients received either cisplatin and etoposide (PE: 30 mg/m^2^ cisplatin on days 1–3 and either 100 mg/m^2^ etoposide on days 1–5 or 100 mg/m^2^ etoposide on days 1–3) or carboplatin and etoposide (CE: carboplatin AUC 5 or 300 mg/m^2^ on day 1 and either 100 mg/m^2^ etoposide on days 1–5 or etoposide 100 mg/m^2^ on days 1–3). In our center, postoperative radiotherapy (PORT) was usually conducted to patients with lymph node metastasis. In this series, 55 patients were given PORT, 49 patients of them were staged as N2 and the remaining 6 patients were staged as N1. The reasons of patients with lymph node metastasis not accept PORT were as follows: patients refusal, poor lung function or low Karnofsky performance status (KPS) after surgery. Chemotherapy and radiotherapy after surgery were given sequentially to 43 patients and concurrently to 12 patients. TRT was administered by three-dimensional conformal radiotherapy technique. The clinical target volume (CTV) included the bronchial stump, ipsilateral hilum, and adjacent mediastinal lymph nodes, and the planning target volume included the CTV with a 1-cm margin. Radiation was delivered with megavoltage linear accelerators. A total dose of 50–60 Gy was administered with 1.8–2 Gy per fraction for 5 days a week.

### Statistical analysis

Overall survival (OS) was measured from the date of surgery to the date of death from any cause or the last known date that the patient was alive. Time to event of BM was monitored from the date of surgery to the date of BM or to the date of last follow-up if no BM occurred. The actuarial risk of developing BM and survival were estimated by the Kaplan–Meier method. The log-rank test was used to compare the difference between groups. Multivariate analyses for BM and OS were performed using Cox regression and a backward-forward stepwise method was selected. Two-sided *p* values < 0.05 were considered statistically significant.

## Results

### Patient characteristics

Records of 126 patients were included and analyzed in this study. Patient characteristics are summarized in Table 1. The median follow-up period for all patients was 56.0 months (range, 30.4–96.8 months). The median age was 55 years (range, 34–74 years).

**Table 1:** **Clinical features of patients with resectable SCLC**

Characteristic	No.	%
**Gender**		
Male	101	80.2
Female	25	19.8
**Age (years)**		
Range	37–74	
Median	55	
<65	91	72.2
≥65	35	27.8
**KPS score**		
≥80	80	64.0
<80	46	36.0
**Smoking status**		
Yes	72	57.1
No	54	42.9
**P-stage**		
I	32	25.4
II	33	26.2
III	61	48.4
**LVI**		
Yes	30	23.8
No	96	76.2
**PORT**		
Yes	55	43.7
No	71	56.3
**No. Cycles of ChT**		
<4	21	16.7
≥4	105	83.3

*Abbreviations*: SCLC = small cell lung cancer; KPS = Karnofsky performance status; P-stage = pathologic stage; LVI = lymphovascular invasion; PORT = postoperative radiotherapy; ChT = chemotherapy.

### Factors predictive of the OS

**Figure 1:**
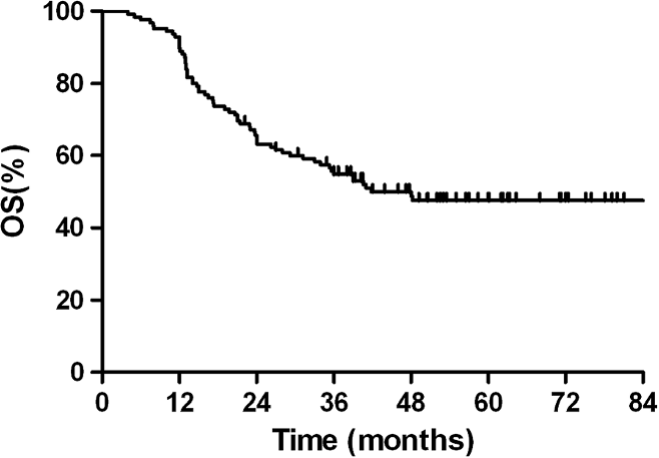
**Overall survival curve for 126 patients with completely resected small cell lung cancer.**

**Figure 2:**
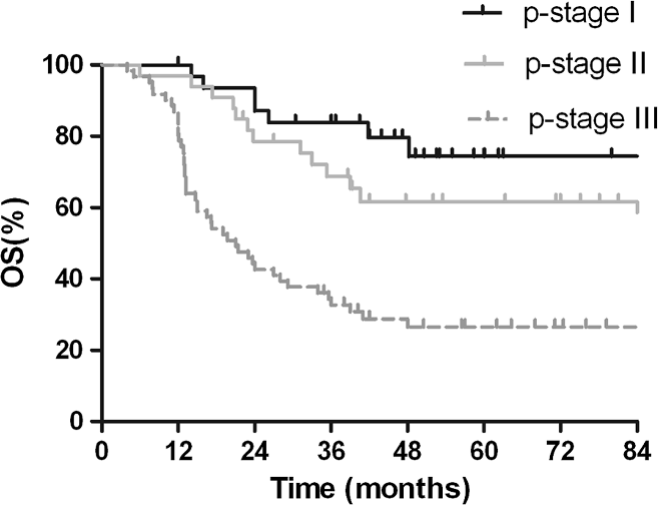
**Comparison of overall survival of patients with surgically resected small cell lung cancer by pathologic stage.**

**Figure 3:**
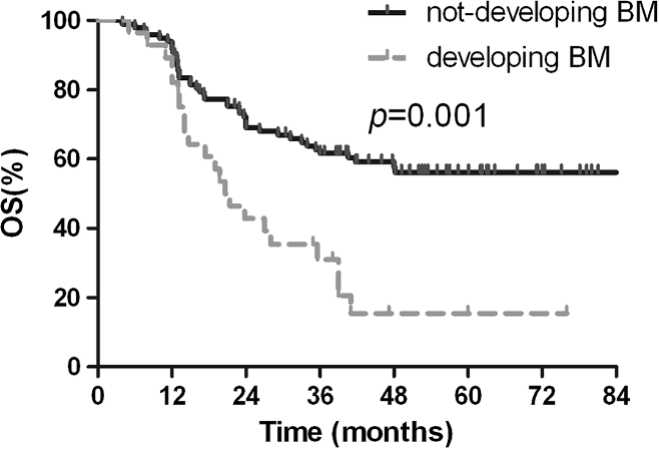
**Comparison of overall survival between patients based on development of brain metastases.**

Median survival time for this patient population was 48.0 months. The OS rates at 2 years and 5 years were 63.2% and 47.8%, respectively (Figure 1). The clinical and pathological factors evaluated to determine their prognostic value for OS are summarized in Table 2. Univariate analysis revealed that pathologic(p) -stage and development of BM were significant factors that correlated with survival rate. The 2-year and 5-year survival rates were 87.1% and 74.4%, respectively, for patients with p-stage I disease; 78.6% and 61.7%, respectively, for patients with p-stage II disease; and 42.6% and 26.6%, respectively, for patients with p-stage III disease (*p* = 0.001; Figure 2). Additionally, survival was significantly longer in the patients who did not develop BM than in patients who developed BM, with a 2-year and 5-year OS of 69.0% and 56.2% versus 42.9% and 15.5%, respectively (*p* = 0.001; Figure 3). Multivariate analysis revealed that age ≥ 65 years (hazard ratio [HR] = 1.798, *p* = 0.040), pathologic stage (HR = 2.093, *p* = 0.001), and developing BM (HR = 2.092, *p* = 0.031) were independent prognostic factors for OS.

**Table 2:** **Univariate and multivariate analysis of the effect of prognostic factors on OS in patients with resectable SCLC**

Factors	Univariate analysis				Multivariate analysis		
	2y-OS %	5y-OS %	***X*** ^***2***^	***p***	HR	95% CI	***p***
Gender							
Male	63.0	48.4					
Female	64.0	43.6	0.015	0.901			
Age, years							
<65	64.8	53.3					
≥65	58.9	32.2	3.079	0.079	1.798	1.027 ~ 3.148	0.040
KPS score							
≥80	71.5	56.9					
<80	61.5	42.3	3.487	0.062	1.149	0.631 ~ 2.092	0.649
Smoking status							
Yes	62.1	52.0					
No	64.7	42.3	0.284	0.594			
P-stage							
I	87.1	74.4					
II	78.6	61.7					
III	42.6	26.6	28.70	0.001	2.093	1.399 ~ 3.132	0.001
LVI							
Yes	48.6	34.1					
No	67.6	51.8	3.358	0.067	0.935	0.507 ~ 1.723	0.829
PORT							
Yes	64.7	43.6					
No	63.0	48.2	0.028	0.866			
Cycle of ChT							
<4	61.9	57.1					
≥4	63.5	46.0	0.221	0.638			
Brain metastasis							
Yes	42.9	15.5					
No	69.0	56.2	14.65	0.001	2.092	1.049 ~ 4.170	0.031

*Abbreviations*: OS = overall survival; SCLC = small cell lung cancer; 2y-OS = overall survival rate at 2 years; 5y-OS = overall survival rate at 5 years; HR = hazard ratio; CI = confidence interval KPS = Karnofsky performance status; P-stage = pathologic stage; LVI = lymphovascular invasion; PORT = postoperative radiotherapy; ChT = chemotherapy.

### Factors predictive of BM

Twenty-eight patients (28/126, 22.2%) developed BM. The time for development of BM ranged from 1.3 months to 75 months, with a median time to development of BM of 10.9 months,which was calculated from the time of diagnosis. The incidence of BM was 9.4% (3/32) in patients with p-stage I disease, 18.2% (6/33) in patients with p-stage II disease, and 31.1% (19/61) in patients with p-stage III disease.

**Figure 4:**
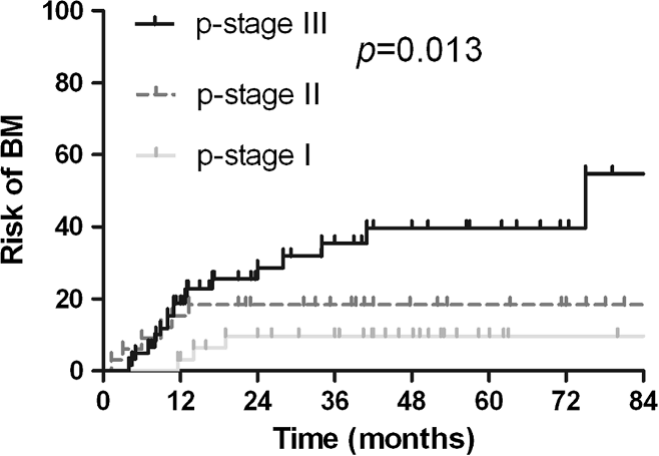
**The actuarial risk of developing brain metastases in patients with different pathologic stage of completely resected SCLC.**

**Figure 5:**
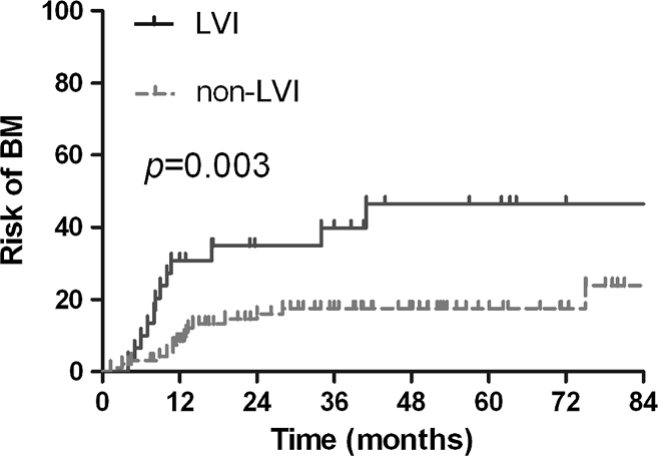
**The actuarial risk of developing brain metastases based on the presence or absence of lymphovascular invasion.**

The actuarial risk of developing BM in all patients at 1 year and 3 years was 13.8% and 23.0%, respectively. The clinical and pathological factors evaluated to determine their prognostic value for the actuarial risk of developing BM are summarized in Table 3. The risk of developing BM was associated with pathologic stage. The actuarial risk of developing BM at 1 year and 3 years was 3.1% and 9.7%, respectively, in patients with p-stage I disease; 15.4% and 18.5%, respectively, in patients with p-stage II disease; and 18.8% and 35.4%, respectively, in patients with p-stage III disease (*p* = 0.013, Figure 4). Lymphovascular invasion (LVI) was also associated with BM. The risk of developing BM at 1 year and 3 years in patients with LVI were 30.8% and 39.9%, respectively, which is significantly higher compared with 9.8% and 17.5% respectively in patients without LVI (*p* = 0.003; Figure 5).

**Table 3:** **Factors associated with actuarial risk of developing BM for patients with resectable SCLC**

Factors	Univariate analysis				Multivariate analysis		
	1y	3y	3y	***p***	HR	95% CI	***p***
Gender							
Male	14.3	23.0					
Female	12.0	23.6	0.04	0.906			
Age, years							
<65	14.5	22.4					
≥65	12.0	26.3	0.063	0.802			
KPS score							
≥80	10.6	17.3					
<80	16.0	31.5	1.207	0.272			
Smoking status							
Positive	15.9	24.7					
Negative	11.2	20.9	0.341	0.559			
P-stage							
I	3.1	9.7					
II	15.4	18.5					
III	18.8	35.4	8.621	0.013	2.013	1.135 ~ 3.569	0.017
LVI							
Yes	30.8	39.9					
No	9.8	17.5	8.943	0.003	1.924	1.002 ~ 3.291	0.039
PORT							
Yes	17.6	33.5					
No	13.2	21.3	3.351	0.067	0.825	0.329 ~ 2.064	0.680
Cycle of ChT							
<4	10.0	17.5					
≥4	14.5	24.2	0.240	0.624			

*Abbreviations*: SCLC = small cell lung cancer; 1y = actuarial risk of developing BM at 1 year; 3y = actuarial risk of developing BM at 3 years; HR = hazard ratio; CI = confidence interval; KPS = Karnofsky performance status; P-stage = pathologic stage; LVI = lymphovascular invasion; PORT = postoperative radiotherapy; ChT = chemotherapy.

Multivariate analysis indicated that pathologic stage (HR = 2.013, *p* = 0.017) and LVI (HR = 1.924, *p* = 0.039) were independent factors associated with increased risk of developing BM. Based on the results of the multivariate analysis, we analyzed the effect of these two independent high-risk factors affecting the incidence of BM. In patients with p-stage II disease and LVI (n = 11), the 1-year and 3-year actuarial risk of developing BM was 29.3% and 29.3%, respectively. In patients with p-stage II disease and no LVI (n = 22), the 1-year and 3-year actuarial risk of developing BM was 9.1% and 13.6%, respectively. In patients with p-stage III disease and LVI (n = 17), the 1-year and 3-year actuarial risk of developing BM was 35.3% and 52.1%, respectively. In patients with p-stage III disease and no LVI (n = 44), the 1-year and 3-year actuarial risk of developing BM was 12.2% and 27.5%, respectively. The differences among the groups were statistically significant (*p* = 0.015). We did not analyze patients with p-stage I disease because only two patients with p-stage I disease had LVI.

## Discussion

Chemotherapy combined with TRT is the standard treatment for the management of limited-stage (LS) SCLC. Nevertheless, the role of surgery as a part of multimodality treatment remains controversial. The NCCN guidelines recommend lobectomy and mediastinal lymph node dissection followed by ChT only for patients who are classified with clinical stage T1-2 N0 disease and who have negative pathological mediastinal staging after mediastinoscopy [10]. However, if the neoplasm was peripherally located and clinically diagnosed as malignant by experienced radiologists, and the result of transthoracic needle biopsy or bronchoscopy-based biopsy was negative, then surgery was still performed. In addition, surgery occasionally was performed at the discretion of the surgeons and/or the patients’ preference.

A favorable role for surgery in LS-SCLC treatment was alluded to in a Medical Research Council study [11]. However, in this clinical trial, only 34 of the 71 patients (48%) scheduled to undergo surgery actually underwent surgery. Not surprisingly, surgery was not found to improve OS in this trial. A randomized Lung Cancer Study Group study reached a similar conclusion [12]. However, patients with T1N0 disease were excluded from this trial, and thus possibly explained the low survival of patients with early stage SCLC. The role of surgery was re-evaluated after the recent introduction of the TNM staging system. A matched-pair analysis comparing 67 patients who underwent surgery followed by adjuvant ChT and 67 patients who received conventional non-surgical management revealed a 5-year OS of 27% for the surgical group and 4% for the matched non-surgical cohort. Subset analysis confirmed significantly longer survival following surgery for T1–2 and N0–1 categories [13]. Several trials have demonstrated that surgery with adjuvant ChT results in a favorable survival rate for p-stage I-III disease with a 5-year OS of 31%–71% for patients with p-stage I disease [14–18]. The data from the Surveillance, Epidemiology, and End Results registry in 2010 was used to analyze the role of surgery in patients with localized disease (T1-T2Nx-N0) or regional disease (T3-T4Nx-N0). Of the 14,179 patients identified, 863 underwent surgical resection. Surgery was associated with improved survival for both localized disease and regional disease. The 5-year OS for patients who received surgery for localized disease was 44.8% compared to an OS of just 13.7% for the non-surgery group (*p* < 0.001). In addition, the 5-year OS for patients with regional disease was 26.3% compared to an OS of 9.3% for the non-surgery group (*p* < 0.001). Furthermore, the OS for patients with lymph node involvement was also improved in the surgery group [19]. Our study demonstrates that the 5-year OS of patients with completely resected SCLC was 47.8%, while the 5-year survival rates for p-stage I, p-stage II, and p-stage III disease were 74.4%, 61.7%, and 26.6%, respectively, which are similar to the rates reported by others.

Additionally, in this series, most patients with lymph node metastasis were conducted with postoperative radiotherapy (PORT), which could improve the local control and give survival benefit to the subgroup.

The positive role of PCI in patients with limited SCLC and extensive SCLC who achieve a complete response (CR) to ChT was established in 1999 by a meta-analysis that included data from seven randomized prospective studies comparing PCI with no PCI after a CR [6]. The 3-year survival rate was 20.7% for patients who received PCI compared with 15.3% for those who did not receive PCI (*p* = 0.01). The disease-free survival of patients who received PCI was also improved. The role of PCI on SCLC was established on the basis of the two-stage system and the treatment modality of ChT and/or TRT. However, the use of PCI after combined-modality treatment with surgery for resectable LS-SCLC has not been investigated sufficiently. The analysis of surgical SCLC could provide evidence regarding the value of PCI since these specimens permit accurate pathological diagnosis and TNM staging. As far as we are aware, only four studies have evaluated the frequency of BM after surgery for LS-SCLC. In the study conducted by Gong et al., the frequency of BM in patients with p-stage I, II, and III diseases were 6.25% (2/32), 28.2% (11/39), and 29.1% (16/55), respectively [20]. One Japanese multi-institutional phase II study (JCOG9101) has reported that the overall incidence of BM was 15% (9/61) in patients with surgically resected SCLC, but only 11% (4/35) in patients with p-stage I disease compared to 19.2% in patients with p-stage II or stage III disease [15]. Nakamura et al. analyzed the frequency of BM as a first relapse site and found a 7% (2/30) BM rate for patients with p-stage I disease, 25% (3/12) for patients with p-stage II SCLC, and 27% (7/26) for patients with p-stage III [21]. Ogawa et al. also reported the frequency of BM, but only 28 patients were included in the study [22]. The combined results from these four studies revealed that BM as the first complication developed in only 7.2% (8/111) of patients with p-stage I SCLC, while BM developed in 30.3% (20/66) of patients with p-stage II and 24.5% (26/106) of patients with p-stage III.

In our study, we found that the incidence of BM was only 9.4% (3/32) in patients with p-stage I disease. Similarly, the results of the four studies discussed above also suggest that PCI maybe not necessary for patients with p-stage I SCLC. Only one retrospective study has suggested a survival benefit is conferred by the use of PCI in surgically managed patients with p-stage I and II disease. In this study, only 39 cases with resected pT1–2 N0–1 M0 LS-SCLC were included, BM-free survival (*p* = 0.01) and OS (*p* = 0.01) were improved in patients who received PCI [23]. To summarize, the incidence of BM in patients with p-stage I SCLC was less than 10% and the evidence of survival benefit of PCI for this subgroup patients was in shortage, so PCI should be considered cautiously for surgically managed patients with p-stage I SCLC and deserve further study.

## Conclusion

Patients with completely resected p-stage II/III SCLC and LVI are at the highest risk for BM.
